# Integrating proteomics and explainable artificial intelligence: a comprehensive analysis of protein biomarkers for endometrial cancer diagnosis and prognosis

**DOI:** 10.3389/fmolb.2024.1389325

**Published:** 2024-06-03

**Authors:** Seyma Yasar, Fatma Hilal Yagin, Rauf Melekoglu, Luca Paolo Ardigò

**Affiliations:** ^1^ Department of Biostatistics, and Medical Informatics, Medicine Faculty, Inonu University, Malatya, Türkiye; ^2^ Department of Obstetrics and Gynecology, Faculty of Medicine, Inonu University, Malatya, Türkiye; ^3^ Department of Teacher Education, NLA University College, Oslo, Norway

**Keywords:** machine learning, explainable artificial intelligence, endometrium cancer, proteomic, biomarker

## Abstract

Endometrial cancer, which is the most common gynaecological cancer in women after breast, colorectal and lung cancer, can be diagnosed at an early stage. The first aim of this study is to classify age, tumor grade, myometrial invasion and tumor size, which play an important role in the diagnosis and prognosis of endometrial cancer, with machine learning methods combined with explainable artificial intelligence. 20 endometrial cancer patients proteomic data obtained from tumor biopsies taken from different regions of EC tissue were used. The data obtained were then classified according to age, tumor size, tumor grade and myometrial invasion. Then, by using three different machine learning methods, explainable artificial intelligence was applied to the model that best classifies these groups and possible protein biomarkers that can be used in endometrial prognosis were evaluated. The optimal model for age classification was XGBoost with AUC (98.8%), for tumor grade classification was XGBoost with AUC (98.6%), for myometrial invasion classification was LightGBM with AUC (95.1%), and finally for tumor size classification was XGBoost with AUC (94.8%). By combining the optimal models and the SHAP approach, possible protein biomarkers and their expressions were obtained for classification. Finally, EWRS1 protein was found to be common in three groups (age, myometrial invasion, tumor size). This article’s findings indicate that models have been developed that can accurately classify factors including age, tumor grade, and myometrial invasion all of which are critical for determining the prognosis of endometrial cancer as well as potential protein biomarkers associated with these factors. Furthermore, we were able to provide an analysis of how the quantities of the proteins suggested as biomarkers varied throughout the classes by combining the SHAP values with these ideal models.

## 1 Introduction

Endometrial cancer (EC) is one of the most common gynecological malignancies in developed countries, with more than 382.000 new cases diagnosed worldwide in 2018 (Bray et al., 2018) and in the United States alone, 66.570 new cases were recorded in 2021 (Siegel et al., 2021). More than 300.000 new cases are diagnosed each year, accounting for approximately 8.2% of the cancer incidence in women worldwide. Its incidence varies by region and increases with increasing life expectancy (Lortet-Tieulent et al., 2018). Age, tumor grade, and depth of myometrial invasion are known prognostic factors for EC. The depth of myometrial invasion is an independent prognostic factor for lymph node metastasis. While the extent of the tumor can be determined by diagnostic curettage, the depth of myometrial invasion can be determined definitively by postoperative pathological examination of the specimen. Despite this, knowing the presence and depth of myometrial invasion in the preoperative period is important for treatment planning (Dane and Bakir, 2019). The most widely used grading system for EC is the Federation of Gynecology and Obstetrics (FIGO) grading system, which consists of three degrees. Mainly, those with a good prognosis are grade I, those with a moderate prognosis are grade II and often poor prognosis, and those associated with an increased risk of myometrial invasion and lymph node metastasis are grade III lesions (Lewin, 2011). According to FIGO, the probability of recurrence is 10%–20% in stages I-II and 50%–70% in stages III-IV (Tejerizo-García et al., 2013). Postmenopausal bleeding, thought of as blood in the urine by some women, is the most common symptom of EC, although only 10% of women with postmenopausal bleeding have EC. Postmenopausal women with vaginal bleeding undergo a variety of tests to exclude EC, such as transvaginal ultrasound scanning with low specificity and uncomfortable and painful procedures, such as outpatient hysteroscopy and endometrial biopsy (Colombo et al., 2016; Sundar et al., 2017). Therefore, there is an urgent need to develop non- or minimally invasive prognostic tools applicable before surgery for the diagnosis and prognosis of endometrial cancer. Diagnostic tools are needed to determine the number of women at risk of developing endometrial cancer, and prognostic tools are needed to group women with endometrial cancer according to their risk of recurrence before surgery, to recommend and plan the most appropriate treatment, and to avoid over/under treatment. Proteomics is a field that has grown significantly in recent years, thanks to important technological developments that allow accurate and sensitive analysis, and is widely used to identify candidates with biomarker potential in the diagnosis/prognosis of diseases (Aerqin et al., 2022; Li et al., 2022; Messner et al., 2023). In proteomics, liquid chromatography mass spectrometry (LC-MS/MS) based analyzes are frequently used for the analysis of proteins (Kizhakkeppurath Kumaran et al., 2023).

Artificial intelligence, which expresses the simulation of human intelligence in machines, is at the center of many fields (calculation of risk factors for many diseases, diagnosis of cancer, image processing applications, voice recognition, object recognition, etc.) that adopt new information technologies. The ability of intelligent machines to learn, reason, and adapt to solve increasingly complex computational tasks with unprecedented levels of performance has placed artificial intelligence in an important position for the future development of human society. While the first artificial intelligence systems were easily interpretable, the rise of complex black box models such as machine learning and deep neural networks with hundreds of layers and millions of parameters in recent years has led to the need to understand how decisions are made. The main purpose of explainable AI approaches is to help designers uncover a clearly defined set of elements that are important and help them take these elements into account (Arrieta et al., 2020; Aksoy et al., 2022).

Combining proteomics techniques and machine learning, one of the latest developments in high-throughput and impressive technologies, has opened a new era in the discovery and validation of cancer biomarkers (Njoku et al., 2021). The primary aim of this study is to classify tumor size (microscopic vs. macroscopic), myometrial invasion (<10% vs. >10%), age (postmenopausal vs. premenopausal), and grade (Grade I vs. High Grade) using machine learning models. Its secondary purpose is to detect possible protein biomarkers using intelligence methods for clinically interpret the optimal prediction models for tumor size (microscopic vs. macroscopic), myometrial invasion (<10% vs. >10%), age (postmenopausal vs. premenopausal) and grade (Grade I vs. High Grade) in endometrial cancer.

## 2 Materials and methods

### 2.1 Dataset

The dataset used in this study belongs to 20 EC patients with mean age 62.53 (±11.56) and is open access (Jamaluddin et al., 2022). The Inonu University Health Sciences Non-Interventional Clinical Research Ethics Committee approved this study (approval number: 2023/5075). Tumor biopsies from different regions of EC tissue from patients were taken and hen sequential window acquisition of all theoretical fragment ion spectra-mass spectrometry comparative proteomic analysis (SWATH-MS) was performed to reveal the protein content in patient EC tissue. Subsequently, the patients included in the study were reclassified according to grade, myometrial invasion, and tumor size. After the bioinformatics analysis for the myometrial invasion (<10% vs. >10%) group, a total of 101 proteins (79 in the highly invasive (>10%) group and 22 in the less invasive (<10%) group) are detected with different regulation. On the other hand, when comparing patients with Grade I and high-grade EC, a total of 48 proteins (18 in Grade I and 30 in high-grade) were found to have different expression between the two groups. A total of 150 proteins (134 in macroscopic and 16 in microscopic) were found to have different expression when compared with another group of patients with EC, according to tumor size. Finally, a total of 167 proteins (116 in postmenopausal and 8 in premenopausal) were found to have different expression when compared with another group of patients with EC, according to age. Data from patient 13 were not included in the present study (in terms of tumor type homogeneity).

### 2.2 Data preprocessing

Thousands of proteins can be identified and quantified with a single injection of MS-based proteomics technology, a popular approach to profiling protein levels. However, data may be missing value due to some biological (e.g., lack of proteins, protein abundances below the device detection limit) and analytical factors (e.g., sample loss during preparation, incorrect cleavage of peptides) (Karpievitch et al., 2012). Random forest imputation method was used for the missing values in the dataset used in this study (Jin et al., 2021). Missing values per protein was below 5%, and therefore random forest, a multivariate imputation approach, was used. Also used as a variable selection method, least absolute shrinkage and selection operator (LASSO) is the most popular method that reduces overfitting, is suitable for use in data with few observations, and is used to deal with high-dimensional estimation problems (Zhai, 2018) (Details in Supplementary Table S1). In the LASSO method, adding a penalty to the model makes the coefficients reduced to zero or approximately zero. This allows the elimination of unnecessary features in the model by reducing the coefficients of some features completely to zero. Thus, it makes the model simpler and more generalizable. One of the main advantages of LASSO is that redundant or correlated features in the data set can be detected and removed. In this way, the complexity of the model is reduced and the risk of overfitting is reduced. Additionally, how close the coefficients will get to zero can be controlled with lambda (λ), which is the regularization parameter of LASSO. This increases the flexibility of the model (Shi et al., 2021; Wang et al., 2023). Finally, the SMOTE-Tomek sampling method was applied to balance the groups Grade I-High Grade observations and postmenopausal-premenopausal observations. In the SMOTE + Tomek Link analytical method, which is one of the hybrid sampling methods developed as a combination of downsampling and upsampling methods in unbalanced datasets, Synthetic Minority Over-sampling Technique (SMOTE) is used to downsample the observations of the minority class while Tomek Link is used to reduce the observations of the majority class (He and Ma, 2013; Zheng et al., 2015).

### 2.3 Development of prediction models

Three ensemble learning algorithms, LightGBM, XGBoost, and Adaboost, were used to classify endometrial cancer patients into predict tumor size (microscopic vs. macroscopic), mymetrial invasion (<10% vs. >10%) and grade (Grade I vs. High Grade), age (postmenopausal-premenopausal) in EC patients based on protein expressions. Ensemble learning methods are methods that combine more than one learning algorithm or model to represent different features and learning approaches, thus ensuring that the errors of a single model are compensated for by the strengths of the others. Particularly in cancer studies, it is important to bring together different perspectives, given the complexity and heterogeneity of medical data. Additionally, ensemble learning methods and the combination of multiple models ensure that the results obtained are generally more stable and reliable. In many cases, the results of a single model may vary or tend to over fit. However, ensemble learning methods can reduce these risks and provide more consistent results (Zhang et al., 2023; Zolfaghari et al., 2023). A stratified random sampling method was used to divide patients into a training set and a test set at a ratio of 80:20. Grid search method with 5 repeated and 10-fold cross validation was used to optimize the hyperparameters of the machine learning models. The performance of each model was evaluated on the test set and the results were compared. To obtain a more robust performance estimate, avoid reporting biased results, and limit overfitting, the persistence method was repeated 100 times with different random seeds, and the average performance over these 100 times was calculated (Details in Supplementary Table S2). Performance metrics for all models are given with AUC, F1-score, accuracy, specificity, sensitivity (Details in Supplementary Table S3). Considering the performance metrics, the best performing model among the models used in the classification was selected for global explanations.

### 2.4 AdaBoost

AdaBoost algorithm is an ensemble learning method proposed by Freund and Schapire, which can improve the accuracy of weak learner classifiers by changing the distribution of sample weights. The AdaBoost algorithm can transform a poorly qualified classifier that makes erroneous predictions into a new classifier with high classification accuracy. Weak classifiers are integrated with each other by training more than one classifier for the same training set. The AdaBoost algorithm basically changes the distribution of data samples. The new dataset with the modified weights is retrained to obtain a new weak classifier. In the first iteration, the weights of all samples are the same. The weight of misclassified samples increases with each iteration; the weight of the classified samples is reduced and all weights are normalized. In the last step, the best ones from the weak classifiers are selected by voting according to the accuracy values of the prediction classes, and they are combined to form a better classifier by integrating with each other (Y. Zhang et al., 2019).

### 2.5 Extreme gradient boosting (XGBoost)

The Extreme Gradient Boosting (XGBoost) algorithm, an innovative machine learning algorithm, whose article was first published by Tianqi Chen and Carlos Guestrin in 2016, is one of the supervised machine learning algorithms, which is based on the decision tree algorithm and has been frequently used in regression and classification problems recently. The most important features of the XGBoost algorithm are that it can achieve high predictive power, prevent over-learning, manage empty data and do them quickly. XGBoost uses the maximum depth value when building the tree. If the tree created shows excessive downward progress, pruning is performed. While the Gradient Boosting algorithm uses first-order functions to calculate the lost function, XGBoost performs these calculations using second-order functions (Chen and Guestrin, 2016).

### 2.6 Light gradient boosting machines (LightGBM)

The Light Gradient Boosting Machine algorithm can be defined as a fast, high-performance gradient boosting framework using a decision tree engine. Unlike other algorithms with a decision tree infrastructure, the tree grows vertically in the LightGBM algorithm, but horizontally in all other algorithms. It is a structure that can be used especially for classification and sorting. In fact, the underlying and developed idea is the XGBoost method. LightGBM is a gradient boost engine method developed by Microsoft to improve the training time performance of the XGBoost algorithm. LightGBM architecture as the amount of data has increased in recent years, outperforms past and present data science algorithms in terms of speed and is used for classification purposes (Ke et al., 2017).

### 2.7 Explainable artifical intelligence and SHApley additive ExPlanations (SHAP)

Explainable Artificial Intelligence (XAI) is a new field of artificial intelligence research focused on making machine learning algorithms, expressed as black boxes, more interpretable and understandable. XAI algorithms work to generate explanations for its decisions and outputs, providing a higher level of transparency and confidence in artificial intelligences decisions (Stadtler et al., 2022). Within the scope of the study, the SHAP method, which is one of the explainable artificial intelligence methods, was preferred in order to explain the optimal models created to classify the tumor size (microscopic vs. macroscopic), myometrial invasion (<10% vs. >10%), grade (Grade I vs. High Grade), age (postmenopausal-premenopausal). The SHAP method was first developed by Lundberg and Lee (2017), this approach is an artificial intelligence method that explains SHAP values depending on the results regardless of the model. SHAP values indicate the contribution of each feature to the model’s outcome. There is an expected output value from the model for the trained data set. This value is called the base value. The contribution of SHAP values to the model shows how far the model deviates from this base value. Features that contribute more are considered features that are important to the model. These contributions can be both negative and positive. Absolute SHAP values indicate the importance of features, while the average of absolute SHAP values for all results indicates general importance values (Lundberg and Lee, 2017).

### 2.8 Statistical analysis

Quantitative data are summarized as mean ± standard deviation and median (minimum-maximum). The normal distribution was evaluated with the Shapiro-Wilk test. The existence of a statistically significant difference between the categories of the output variable in terms of proteins was examined with the independent sample *t*-test and Mann-Whitney U. p < 0.05 values were considered statistically significant. IBM SPSS Statistics for Windows version 26.0 software (George and Mallery, 2019) and GraphPad Prism 9.4.1 software were used for all statistical analyzes and graphical representations, respectively (Details in Supplementary Tables S4.1–S4.5).

## 3 Results

After the LASSO variable selection method, which was applied to prevent over-learning in the models to be created with the dataset of proteomic data obtained from tumor tissue biopsy of EC patients, 14 proteins were selected for tumor size, 17 proteins were selected for myometrial invasion, and finally 9 proteins for Grade I vs. High Grade (Details are in Supplementary Table S1). The AUC, accuracy, sensitivity, specificity, and F1-Score performance metrics for the models created for tumor size (microscopic vs. macroscopic), mymetrial invasion (<10% vs. >10%), grade (Grade I vs. High Grade), and age (postmenopausal-premenopausal) are presented in Table 1.

**TABLE 1:** Results on predictive performance of machine learning models in tumor size, myometrial invasion, tumor grade, and age results.

a) Tumor size (Macroscopic vs. Microscopic)					
Model	Accuracy	Sensitivity	Specificity	F1-score	AUC
XGBoost	91.8	100	79.2	93.7	94.8
AdaBoost	88.5	94.9	77.3	91.4	91.9
LightGBM	90.2	97.4	78.3	92.5	93.4

b) Myometrial Invasion (>10% vs. <10%)					
Model	Accuracy	Sensitivity	Specificity	F1-Score	AUC
XGBoost	91.5	92.1	90.5	93.3	92.2
AdaBoost	91.5	94.4	87.0	93.2	90.7
LightGBM	94.9	97.2	91.3	95.9	95.1

c) Grade (Grade I vs. High Grade)					
Model	Accuracy	Sensitivity	Specificity	F1-Score	AUC
XGBoost	97.3	97.3	97.3	97.3	98.6
AdaBoost	90.5	89.5	91.7	90.7	94.4
LightGBM	93.2	92.1	94.4	93.3	96.2

d) Age (Postmenopausal vs. Premenopausal)					
Model	Accuracy	Sensitivity	Specificity	F1-Score	AUC
XGBoost	98.8	100	97.6	98.8	98.8
AdaBoost	96.3	97.4	95.3	96.2	96.3
LightGBM	97.6	97.5	97.6	97.5	97.6

AUC: Area under the ROC, curve.

The XGBoost model had the highest performance metric with 94.8 AUC and 91.8 accuracy in classifying tumor size. In classifiying myometrial invasion, the LightGBM model had the highest performance metric, with 96.1 AUC and 93.4 accuracy. Similarly, in the classification of Grade I and Grade II, the XGBoost model has the highest performance metric with 95.4 AUC and 91.9 accuracy. On the other hand, when the performance metrics of the models created in the classification of tumor stages are examined, the XGBoost model had the highest performance with 98.6 AUC and 97.3 accuracy value for Grade I vs. High Grade. Finally, considering the performance metrics of the models created in age classification, the XGBoost model had the highest performance with an AUC of 98.8 and an accuracy value of 98.8 for postmenopausal and premenopausal.

**FIGURE 1:**
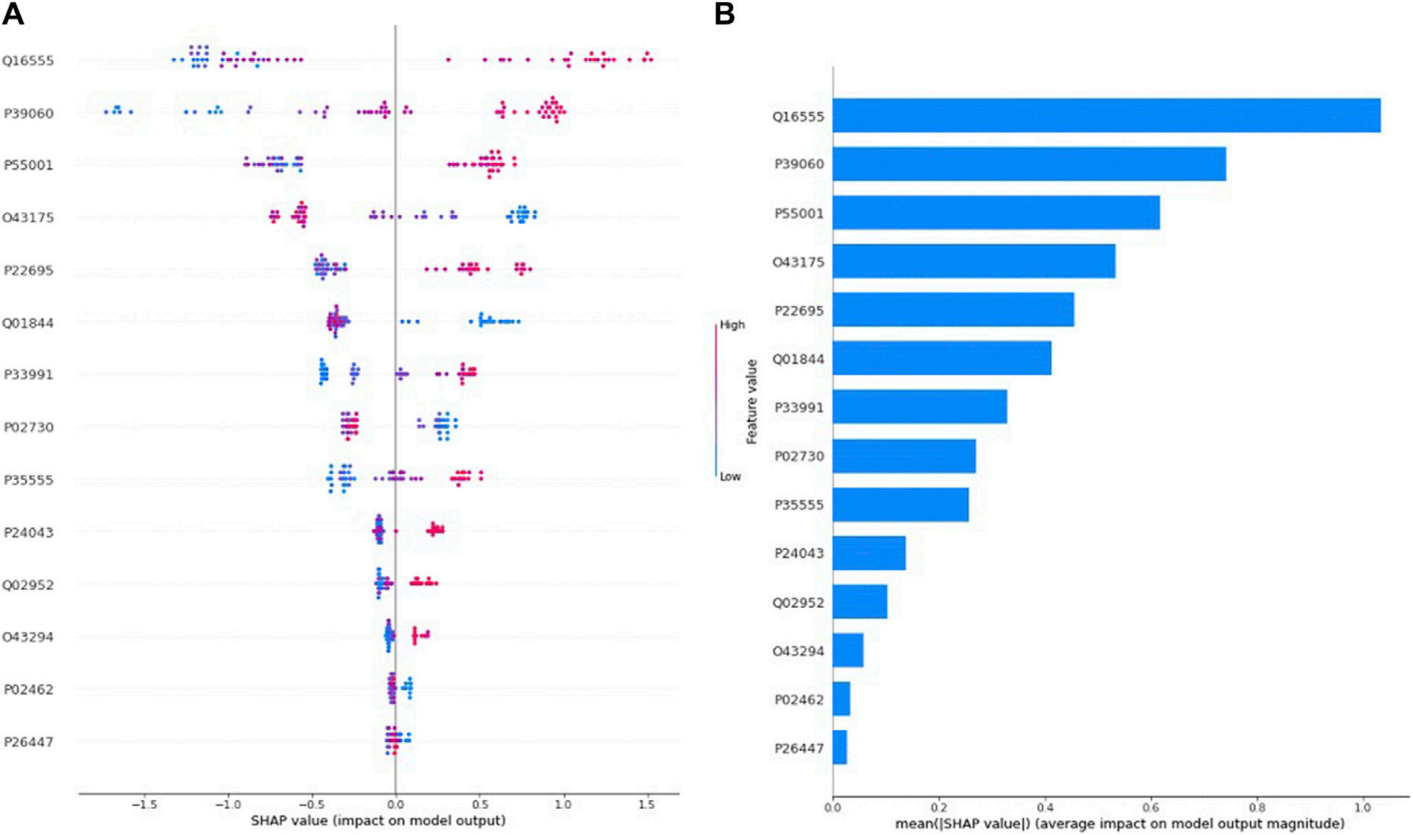
**(A)** Global SHAP annotations for tumor size prediction of the XGBoost model. Horizontal positions reflect the effect of proteins on model output. Colors indicate whether protein is high (red) or low (blue) for a particular patient. A positive SHAP value indicates a positive contribution to the output, and a negative SHAP value indicates a negative contribution to the output. **(B)** Graphs of protein importance based on the mean SHAP values of the XGBoost model in predicting tumor size. The graph shows an order of importance for proteins according to their collective absolute SHAP values.

Figure 1A presents the SHAP value plot for the tumor size optimal model XGBoost, as an alternative global interpretation scheme. These bee swarm plots express positive/negative relationships with the target variable in addition to the significance of the predictors. Each point on the charts corresponds to a sample in the data. Colors represent the relative values of variables. In estimation of tumor size, blue and red color denote low and high values, respectively, for biomarker candidate proteins. Therefore, it was determined that the high values of the proteins with the accession code Q16555, P39060, and P55001 as well as the low values of the proteins with the accession code Q43175, Q01844 increased the risk of macroscopic tumor size in EC patients. Figure 1B protein importance plots list in descending order the proteins most important for the optimal model XGBoost in the tumor size prediction task based on their collective SHAP values. The length of each bar represents the average of the absolute SHAP values for the protein(s) of interest. According to Figure 1B, the top five proteins most important in predicting tumor size are those with the accession code Q16555, P39060, P55001, Q43175, and P22695, respectively.

**FIGURE 2:**
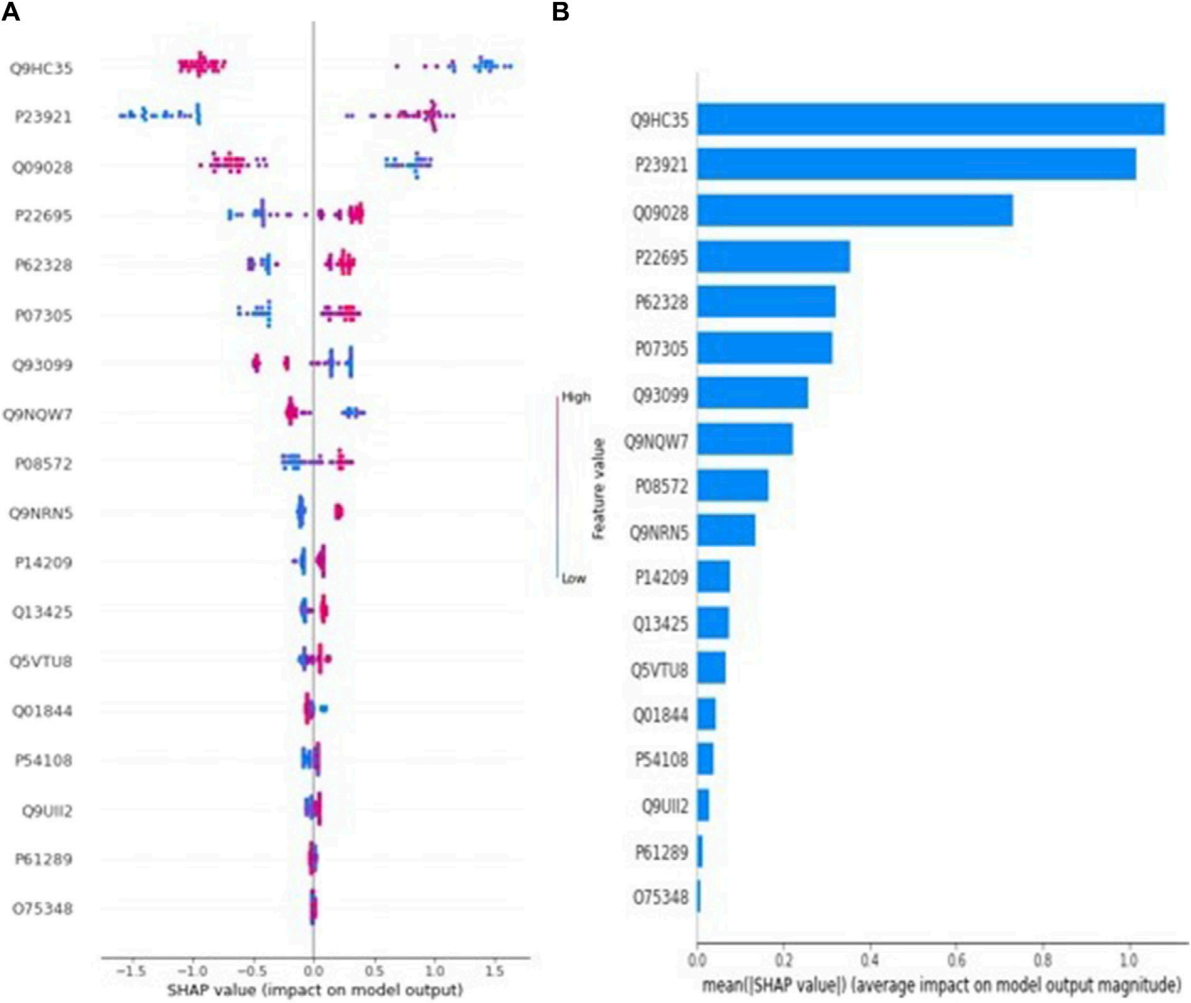
**(A)** Global SHAP annotations for myometrial invasion prediction of the LightGBM model. Horizontal positions reflect the effect of proteins on model output. Colors indicate whether protein is high (red) or low (blue) for a particular patient. A positive SHAP value indicates a positive contribution to the output, and a negative SHAP value indicates a negative contribution to the output. **(B)** Graphs of protein importance based on the mean SHAP values of the LightGBM model in predicting myometrial invasion. The graph shows an order of importance for proteins according to their collective absolute SHAP values.

Similarly, Figure 2A presents the SHAP value plot for the myometrial invasion optimal model LightGBM. Considering Figure 2A, it can be said that low values of proteins with accession code Q9HC35, Q09028, Q9NQW7 and high values of proteins with accession code P23921, P07305 increase the risk of highly invasive of mymetrial invasion in EC patients. On the other hand, according to Figure 2B, the five most important proteins in estimating myometrial invasion are those with accession codes Q9HC35, P23921, Q09028, Q9NQW7, and P07305, respectively.

**FIGURE 3:**
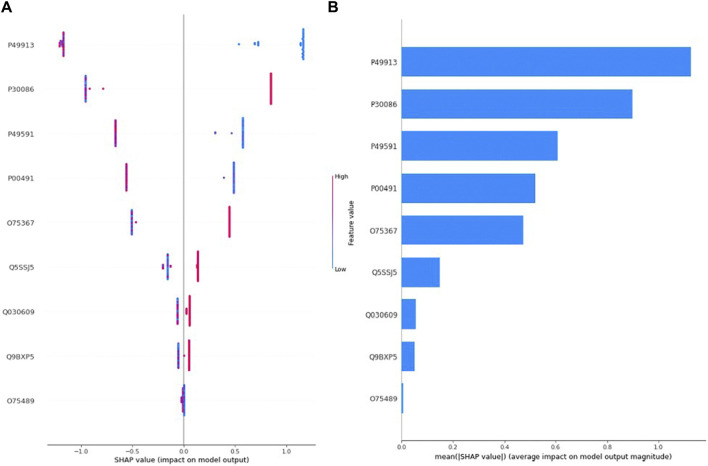
**(A)** Global SHAP annotations for Grade 1 vs. High Grade prediction of the XGBoost model. Horizontal positions reflect the effect of proteins on model output. Colors indicate whether protein is high (red) or low (blue) for a particular patient. A positive SHAP value indicates a positive contribution to the output, and a negative SHAP value indicates a negative contribution to the output. **(B)** Graphs of protein importance based on the mean SHAP values of the XGBoost model in predicting Tumor Grade 1 vs. High Grade. The graph shows an order of importance for proteins according to their collective absolute SHAP values.

Figure 3A depicts the SHAP value plot for the tumor grade (Grade I-High Grade) optimal model XGBoost. Regarding Figure 3A, it can be said that low values of proteins with accession P49913, P49591, P00491 and high values of proteins with accession P30086, O75367 increase the risk of high grade in EC patients. On the other hand, according to Figure 3B, the five most important proteins in predicting tumor grade are proteins with accession P49913, P30086, P49591, P00491, and O75367, respectively.

**FIGURE 4:**
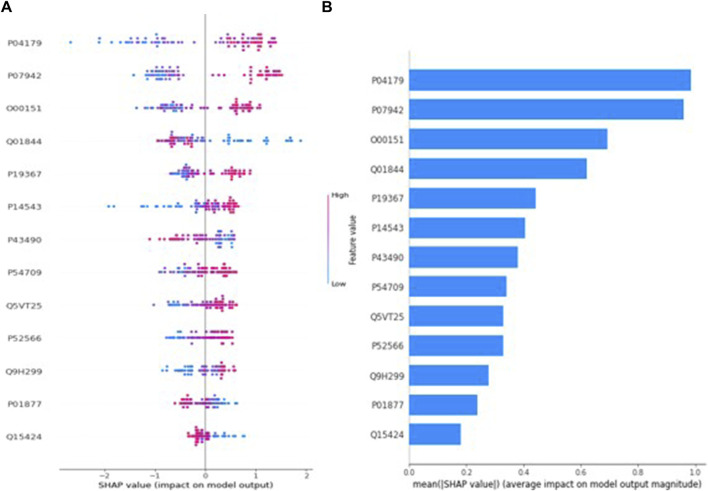
**(A)** Global SHAP annotations for postmenopausal and premenopausal prediction of the XGBoost model. Horizontal positions reflect the effect of proteins on model output. Colors indicate whether protein is high (red) or low (blue) for a particular patient. A positive SHAP value indicates a positive contribution to the output, and a negative SHAP value indicates a negative contribution to the output. **(B)** Graphs of protein importance based on the mean SHAP values of the XGBoost model in predicting postmenopausal vs. premenopausal. The graph shows an order of importance for proteins according to their collective absolute SHAP values.

Similarly, Figure 4A describes the SHAP value plot for the age (postmenopausal-premenopausal) optimal model XGBoost. Regarding Figure 4A, it can be said that low values of proteins with accession Q01844 and high values of proteins with accession P04179, P07942, O00151, P19367 increase the risk of premenopausal in EC patients. However, in compliance with Figure 4B, the five most important proteins in predicting age are proteins with accession P04179, P07942, O00151, Q01844, P19367, respectively.

## 4 Discussion

EC continues to be the prevalent gynecological cancer worldwide, despite ongoing efforts to discover new prevention, diagnostic, and treatment methods. EC is a type of cancer that can relapse or spread to different regions even in the early stage. Accordingly, unfortunately, the mortality and morbidity rates linked to this cancer are it continues to increase. As in every cancer disease, predicting the prognosis in endometrial cancer is one of the biggest problems. Current treatment options are limited to surgery and additional therapies. Although the prognosis of endometrial cancer is determined by the final stage of FIGO, studies have shown that factors such as tumor grade, tumor size, and myometrial invasion affect the postoperative situation (Morice et al., 2016; Eakin et al., 2023). Therefore, a deep understanding of the molecular mechanisms, especially at the protein level, is crucial to finding new ways to treat endometrial cancer.

### 4.1 Protein biomarker for age (premenopausal-postmenopausal) via XGBoost

In this study, three different machine learning models (XGBoost, AdaBoost, and LightGBM) were used to classify proteomic data in terms of age, tumor size, myometrial invasion and tumor grade, aiming to detect unique protein expressions of tissue samples from 20 EC patients. Considering the performance metrics of three different classification models for age, which is a risk factor for EC and grouped as premenopausal and postmenopausal in the study, XGBoost have the highest accuracy (98.8%), sensitivity (100%), specificity (97.6%), F1-Score (98.8%), and AUC (98.8%) values. According to the SHAP method results applied to the XGBoost model to help clinicians better understand the model’s decision making process, 5 possible protein biomarker candidates in postmenopausal and premenopausal classification in endometrium cancer patients can be said P04179, P07942, O00151, Q01844, P19367. The expression of superoxide dismutase (P04179), which is directly related to tumor progression, invasion and angiogenesis and is synthesized by the SOD2 gene, is higher in premenopausal EC patients than in postmenopausal EC patients (*p* < 0.001). It was reported that the protein in question was expressed at a higher rate in endometrial cancer patients in the study conducted by Fuzi et al. (2018).

### 4.2 Protein biomarker for myometrial invasion (<10%–>10%) via LightGBM

The depth of myometrial invasion is closely linked to the likelihood of lymph node spread and the overall survival of patients with localized uterine cancer. Due to this connection, the FIGO guidelines have categorized patients with early-stage disease into two groups: those with myometrial invasion not exceeding half the total depth of the myometrial wall and those with deeper invasion (Pergialiotis et al., 2023). Within the scope of this study, LightGBM is the one with the highest performance metrics among the three different machine learning models created to classify myometrial invasion. Within the scope of this study, LightGBM is the one with the highest performance metrics, which are accuracy (94.9%), sensitivity (97.2%), specificity (91.3%), F1-Score (95.9%), and AUC (95.1%) values among the three different machine learning models created to classify myometrial invasion. According to the SHAP method after LightGBM, which is the optimal model, Q9HC35, P23921, Q09028, Q9NQW7 and P07305 proteins are recommended to be used as a possible biomarker candidate in the clinic in the classification of myometrial invasion. In this analysis, we found that Q9HC35 (Echinoderm microtubule-associated protein-like 4) was one of the most significant candidate protein for predicting myometrial invasion. This protein primarily serves to modify microtubules, although there is limited proof indicating its role in regulating microtubule dynamics. The specific biochemical mechanisms of its molecular activity remain unexplored. Despite this, interest in these proteins has surged due to the discovery of EML mutations in oncogenic fusions associated with human cancers (Fry et al., 2016). In a specific group of non-small-cell lung carcinoma patients, a genetic abnormality involving echinoderm microtubule–associated protein-like 4 (EML4) has been discovered. This aberration results in the formation of a fusion protein combining the N-terminus of EML4 with the C-terminus of anaplastic lymphoma kinase (ALK). While many tumor types exhibit high microsatellite instability, endometrial cancer stands out as one of the few cancers where this instability is regularly assessed. Although EML4–ALK fusions are observed in only 4%–5% of yearly diagnosed non-small cell lung cancer cases, the remarkable response to ALK inhibitor drugs has led to universal testing for all patients with advanced non-small cell lung cancer (Kurnit et al., 2019). We suggest that, similar to personalized oncology methods, universally assessing the immune microenvironment in all endometrial cancer patients by measuring this protein could be a practical therapeutic approach if it proves to offer a survival advantage.

### 4.3 Protein biomarker for tumor grade (Grade I-High Grade) via XGBoost

Endometrial carcinoma is histopathologically categorized into two primary groups: low grade and high grade. These grades exhibit distinct biological behaviors. The prevalent histological subtype is endometrioid adenocarcinoma. The FIGO grading system for endometrioid endometrial cancer is determined based on the proportion of solid, nonsquamous components. Grades 1, 2, and 3 are characterized by ≤ 5%, 6%–50%, and >50% solid nonsquamous components, respectively. Key prognostic factors for endometrial carcinoma include histologic grade, tumor stage, presence of myometrial invasion, lymphovascular space invasion, and patient age (Rafiee and Mohammadizadeh, 2023). The three machine learning methods used in the study to classify tumor degree are the XGBoost algorithm (accuracy (97.3%), sensitivity (97.3%), specificity (97.3%), F1-Score (97.3%), and AUC (98.6%)). According to the shap values obtained through the XGBoost model, it can be said that P49913, P30086, P49591, P00491, and O75367 proteins are biomarker candidates for tumor grade classification. In our research, we identified a significant protein, P49591 (Seryl-tRNA synthetase), which played a crucial role in predicting tumor grade (Low Grade-High Grade). Aminoacyl-tRNA synthetases (aaRSs) have been recognized as complex proteins with intricate links to human diseases, including cancer. These enzymes consist of 20 cytoplasmic and 19 mitochondrial variants responsible for attaching amino acids to tRNAs, a process essential for protein synthesis. Notably, nearly all cytoplasmic aaRSs, whether associated with the MSC (multi-aminoacyl-tRNA synthetase complex) or existing freely, are involved in regulating various pathways within cells. Dysregulation of these pathways and cellular balance is a prominent characteristic of cancer, and tRNA synthetases have been identified as contributors to tumorigenesis and metastasis through unique mechanisms (Wang et al., 2020). Recent progress in genomics and proteomics studies has revealed unforeseen mutations associated with diseases, as well as changes in expression, secretion, and interactions in human aminoacyl-tRNA synthetases (ARSs) (Kwon et al., 2019). These findings have unveiled previously unknown biological functions of ARSs beyond their traditional role in protein synthesis. The outcomes of this research could highlight the potential of these proteins as a valuable and underexplored resource for new therapeutic targets and agents. This potential could be explored through various approaches, such as directly targeting the catalytic sites, regulating disease-related protein-protein interactions, and developing innovative biologics from the secreted ARS proteins.

### 4.4 Protein biomarker for tumor size (microscopic-macroscopic) via XGBoost

While myometrial invasion, tumor grade, and lymph node metastasis are recognized as individual prognostic factors in endometrial cancer, tumor size holds practical utility in predicting the prognosis of this cancer. The established cutoff values for tumor size align with existing literature, and an increasing body of research suggests a robust correlation with tumor sizes exceeding 20 mm (Jin et al., 2022). Among the machine learning methods used in this study for tumor size classification, XGBoost is the model with the highest performance metrics with accuracy (91.8%), sensitivity (100%), specificity (79.2%), F1-Score (93.7%), and AUC (94.8%). After the SHAP approach applied with the optimal model XGBoost, five possible protein biomarkers that can be recommended as a guide for clinicians in the prognosis of the disease in tumor size classification in patients with EC can be listed as Q16555, P39060, P55001, Q43175, and P22695. In our analysis, we identified P55001 (Microfibrillar-associated protein 2) as a highly significant candidate protein for predicting tumor size. Microfibrillar-associated protein 2 (MFAP2), a component of the extracellular matrix, plays a crucial role in controlling the signal transmission of growth factors. Recent research has indicated that MFAP2, recognized as a reliable prognostic marker in various cancers, is linked to tumor initiation and progression. It might be involved in reshaping the extracellular matrix and regulating processes such as cell growth, programmed cell death, invasion, metastasis, and angiogenesis. However, the exact mechanism of MFAP2 in these tumor-related processes remains unclear (Xu et al., 2022). Considine et al. (2021) utilized extensive data from two genome-wide association studies to pinpoint protein biomarkers associated with ovarian cancer risk in circulating blood. By integrating vast proteomic and genomic datasets, they identified significant and biologically plausible associations between 26 plasma proteins, including MFAP2, and the risk of epithelial ovarian cancer. These associations remained statistically significant after controlling for false discovery rate (Considine et al., 2021). The findings of this research strongly indicate that the identified plasma protein holds promise as a potential biomarker for early detection of this prevalent gynecological cancer. This discovery opens avenues for its practical application in the early diagnosis of the disease, potentially leading to improved screening methods and timely interventions for patients.

Furthermore, RNA-binding proteins (RBPs) are proteins that play critical roles in the post-transcriptional splicing, polyadenylation, mRNA stability, mRNA localisation and translation of RNAs (Mohibi et al., 2019). Different studies have shown that RBPs are aberrant expressed in cancer tissues compared to normal tissues and this expression is associated to patient prognosis (Busa et al., 2007; Janiszewska et al., 2012; Mohibi et al., 2019). In this study, the EWSR1, which is RNA binding protein, was found to be a common protein in age, myometrial invasion and tumor size, which plays an important role in determining the prognosis of endometrial cancer. According to SHAP graphs and statistical analyses, EWSR1 protein expression was higher in patients with postmenopausal age, large size and invasive tumors.

The integration of The Cancer Genome Atlas (TCGA) molecular categorization into the exploration of endometrial cancer biomarkers signifies a crucial progression in the refinement of diagnostic and therapeutic approaches. Our investigation highlights numerous protein biomarkers that hold the potential to augment the existing TCGA molecular classification system by introducing supplementary layers of prognostic insights. Notably, the identified biomarkers, such as the EWRS1 protein, could undergo scrutiny within the framework of TCGA classifications to ascertain their correlation with or predictive capabilities regarding the four established molecular subtypes: POLE ultramutated, microsatellite instability hypermutated, copy-number low, and copy-number high. This alignment could reveal novel insights into subtype-specific pathophysiology and offer avenues for targeted therapeutic interventions.

Additionally, our results have the potential to enhance molecular classification by introducing a proteomic dimension to the genomic markers utilized in TCGA classifications. This enhancement not only enriches comprehension of tumor behavior and progression but also amplifies the accuracy of treatment approaches customized to individual molecular profiles. The capacity of our models to forecast responses to particular treatments based on protein expression patterns indicates that integrating our proteomic data with TCGA’s genomic classifications could heighten the effectiveness of personalized treatment strategies. Recent recommendations underscore the importance of further integrating molecular classifications into treatment determinations; our research reinforces this guidance by suggesting a mechanism to include proteomic perspectives into the molecular typing structure, consequently optimizing therapeutic results for individuals affected by endometrial cancer.

The analysis of proteomics emphasizes the optimistic prognostic significance of POLE mutations, aligning with current knowledge of their ability to enhance AMF/AMFR signal transduction pathways and impact cellular metabolic processes. The presence of POLE mutations has shown a correlation with a positive prognosis, indicating that their identification could be crucial in categorizing patients based on risk and customizing treatment approaches more efficiently. Furthermore, the interplay between POLE mutations and AMF/AMFR signaling pathways may present potential targets for therapeutic intervention in metabolic regulation, potentially steering the progress of innovative treatments. Considering these implications, the functional outcomes of POLE mutations detected in our proteomic analysis offer a more defined understanding of their usefulness in clinical application and their prospective contribution to the advancement of personalized medicine for individuals with endometrial cancer.

The correlation between MSI-H and MMR deficiency in endometrial carcinoma stands as a fundamental aspect in understanding the responsiveness to immune checkpoint inhibitors. The strength of our analysis expands to the discovery of biomarkers capable of predicting responses to immunotherapy, a swiftly progressing domain in the field of gynecologic oncology. Our investigation demonstrates that the existence of MSI-H and MMR deficiencies is linked to an enhanced reaction to immunotherapeutic agents like PD-1 and PD-L1 inhibitors in line with recent clinical trials. This heightened reaction is probably attributable to the elevated mutational load in these neoplasms, leading to the generation of novel antigens, subsequently amplifying the immune system’s capacity to identify and attack malignant cells. The promise of these biomarkers as prognostic instruments for the effectiveness of immunotherapy in patients with endometrial cancer could be a central topic of management. It is essential to contemplate the assimilation of these biomarkers into current therapeutic frameworks, thereby enhancing the selection of patients for immunotherapy and tailoring treatment strategies. Our results propose that incorporating MSI-H and MMR status into clinical decision-making could profoundly influence the therapeutic landscape of endometrial cancer by facilitating the utilization of immunotherapy in a more precise and efficient manner.

The intricate patterns of protein expression associated with the CNL/NSMP (copy-number low or nonspecific molecular profile) and CNH/p53 abnormal (copy-number high or p53 abnormal) categories play a crucial role in influencing the biological behavior and treatment responses in endometrial carcinoma. Our study offers the possibility of identifying new protein biomarkers that may be associated with these distinct molecular profiles. For example, changes in protein expression patterns within the CNH/p53 abnormal category could provide insights into the aggressive nature and poorer prognosis commonly observed in this subtype. Similarly, the CNL/NSMP category could reveal distinctive protein signatures that might guide less aggressive therapeutic approaches or indicate a more favorable prognosis. A thorough exploration of how these protein biomarkers impact tumor characteristics, patient outcomes, and responses to different therapies, such as targeted and hormonal treatments, would offer important context to our results.

The integration of proteomic biomarkers within the clinical management for endometrial cancer offers a promising approach for enhancing treatment personalization and improving prognostic assessments. Our study’s findings elucidate the potential for using specific protein expression profiles to optimize treatment strategies, catering to the unique molecular profile of each patient’s tumor. For example, the presence of proteins associated with aggressive tumor behavior could prompt a more intensive treatment regimen, while the absence of such markers might support a more conservative approach. This personalized therapeutic strategy, which relies on the identification of biomarkers, is especially relevant in the era of targeted therapies and immunotherapies, where accurate patient selection is imperative for favorable results. Moreover, the prognostic value of these biomarkers can significantly impact patient counseling, surveillance strategies, and decisions regarding adjuvant therapies during the posttreatment period. By adopting this approach, a shift can be made from a generalized treatment methodology to a more refined, biomarker-centered model of patient care, potentially enhancing both the quality and specificity of treatment for endometrial cancer patients.

### 4.5 Machine learning methods and explainable artificial intelligence (SHAP) in endometrial cancer

Advances in machine learning techniques, in combination with proteomics, metabolomics and imaging data, offer unique and hopeful perspectives for the discovery of clinically useful biomarkers for the diagnosis and treatment of diseases (Njoku et al., 2021). As in almost all cancer types, machine learning methods have been used with different data types for the diagnosis and diagnosis of endometrial cancer (Stanzione et al., 2021). In previous studies, the estimation performance of classification algorithms created with endometrium cancer with proteomic data ranges from (AUC), 0.80 to 0.92 (Kokol et al., 2023; Njoku et al., 2023). However, the performance of models created for classification in the current study ranges from (AUC), 94.8 to 98.8. Therefore, it can be said that the classification models in question are very successful in classifying endometrium cancer. The employment of machine learning algorithms like XGBoost and LightGBM in the prediction of tumor size, myometrial invasion, and tumor grade in endometrial cancer, as illustrated in our research, demonstrates a potential for enhancing diagnostic accuracy and patient outcomes. An example of this potential lies in the incorporation of these artificial intelligence tools into clinical practice, which could optimize the preoperative evaluation process, potentially diminishing the necessity for invasive diagnostic procedures and enabling more precise therapeutic interventions. Research conducted by Topol has highlighted the capacity of AI to reduce diagnostic inaccuracies by up to 30% when contrasted with conventional approaches, emphasizing the promise of advancing patient safety and treatment efficacy (Topol, 2019). Furthermore, a comparative analysis by Rajkomar et al. (2019) indicates that machine learning algorithms can outperform standard statistical models in predicting health outcomes, suggesting that our models could offer substantial improvements over current diagnostic protocols. To evaluate the feasibility of integrating these models into practice, insights from gynecologic oncologists obtained through structured interviews or surveys would provide invaluable insights into the models’ operational viability and the potential barriers to their adoption in routine clinical settings. By bridging the divide between technological progressions and clinical applications, we can customize AI tools more effectively to address the specific requirements of managing endometrial cancer, thereby improving both prognostic precision and the overall standard of patient care (Huang et al., 2020). The significance of SHAP values lies in their capacity to elucidate the contribution of individual predictors in our predictive models, offering crucial insights for clinical interpretations and interventions. To better illustrate this point, detailed scenarios should be included that showcase how these values influence treatment strategies. For instance, a SHAP analysis revealing a substantial influence of biomarkers like EWRS1 protein on prognosis could prompt clinicians to consider tailored therapies that target the pathways affected by this protein. Similarly, recognizing that factors such as tumor grade exhibit high SHAP values may encourage earlier and more aggressive treatment for patients with high-grade tumors, potentially enhancing outcomes. In a previous investigation, it was noted that SHAP values could offer significant utility in the cytodiagnosis of endometrial cancer, particularly in guiding the selection of patients for endometrial curettage and supplementary diagnostic interventions (Endometrium, 1991). Furthermore, another study highlighted the potential of SHAP values in enhancing diagnostic precision, prognostic assessment, and aiding in tailoring personalized treatment approaches for individuals with endometrial cancer (Banno et al., 2012).

## 5 Limitation

This study has some methodological limitations. One of the key constraints of this research is the lack of validation from an external dataset. Despite the promising results shown by our predictive models within the data collected from the study conducted by Jamaluddin et al. (2022), these results have not been validated against independent external datasets due to the lack of access to appropriate external datasets with sufficient annotation for endometrial cancer prediction. The validation from external sources is essential to evaluate the applicability of the AI models in diverse demographic and clinical settings. Absence of this validation raises uncertainty regarding the models’ suitability for broader populations. This limitation highlights the necessity for future studies to encompass multicenter trials, leading to a more varied data pool. This would ensure the maintenance of predictive accuracy and reliability of the biomarkers across a range of clinical environments. Such researches would play a crucial role in confirming the efficacy of the models and facilitating their integration into clinical practice globally. The second limitation in our study concerns the classification of tumor size and myometrial invasion. The method of the study we used to obtain the proteomic data categorized tumor size as microscopic or macroscopic, which is not consistent with established clinical practice where tumor size is defined as less than or more than 2 cm or not visually identifiable. In addition, the binary classification of myometrial invasion as less than 10% or more than 10% does not correspond to the clinical descriptors used in imaging studies such as MRI or CT scans, where myometrial invasion is typically reported as superficial or deep (involving 1/3 of the myometrium). The use of these non-standardized classifications could limit the direct applicability of our results in routine clinical practice and affect the generalizability of our findings. Future research could benefit from a methodology that uses clinically recognized measures, ensuring greater relevance and usefulness in a clinical setting. The third limitation in our study concerns the classification of tumor size and myometrial invasion. The method of the study we used to obtain the proteomic data categorized tumor size as microscopic or macroscopic, which is not consistent with established clinical practice where tumor size is defined as less than or more than 2 cm or not visually identifiable. In addition, the binary classification of myometrial invasion as less than 10% or more than 10% does not correspond to the clinical descriptors used in imaging studies such as MRI or CT scans, where myometrial invasion is typically reported as superficial or deep (involving 1/3 of the myometrium). The use of these non-standardized classifications could limit the direct applicability of our results in routine clinical practice and affect the generalizability of our findings. Future research could benefit from a methodology that uses clinically recognized measures, ensuring greater relevance and usefulness in a clinical setting.

## 6 Conclusion

In summary, our study integrates a complex proteomic landscape into actionable insights for endometrial cancer using the SHAP method for model interpretation—a novel approach that reveals the complex impact of individual proteins such as EWSR1 and MFAP2 on disease subtyping. This innovative application illustrates how shifts in protein expression contribute to cancer classification, improving clinicians’ understanding and potentially leading to personalized treatments. The identification of such biomarkers is instrumental in improving the precision of molecular profiling in endometrial cancer and provides the basis for future studies to effectively integrate these findings into clinical applications.

## Data Availability

The data analyzed in this study is subject to the following licenses/restrictions: Data used in the study can be requested from the corresponding authors upon appropriate request. Requests to access these datasets should be directed to seyma.yasar@inonu.edu.tr.
