# Supplementary material for: Integrating proteomics and explainable artificial intelligence: a comprehensive analysis of protein biomarkers for endometrial cancer diagnosis and prognosis
Source: Front Mol Biosci. 2024 Jun 3;11:1389325. doi: 10.3389/fmolb.2024.1389325 (PMC11184912; doi:10.3389/fmolb.2024.1389325)
Supplement: Supplementary file 1 [file Table1.DOCX]

Supplementary Material

# Feature Selection

**Supplementary Table 1: Proteins selected on a group basis as a result of LASSO variable selection**

| **Group** | **Proteins Accession** |
| --- | --- |
| Tumor Size (Macroscopic vs Microscopic) | P02730, P35555, O43294, P26447, P55001, P33991, P24043, P02462, Q16555, Q02952, P39060, P22695, O43175, Q01844 |
| Myometrial Invasion (>10% vs <10%) | Q9HC35, P07305, Q13425, Q9UII2, Q09028, O75348, P14209, P61289, Q9NQW7, Q93099, P08572, P22695, P23921, Q9NRN5, P54108, Q5VTU8, P62328 |
| Grade (Grade I vs High Grade) | Q9BXP5, P49591, O75367, Q5SSJ5, P49913, P30086, P00491, O75489, Q030609 |
| Age (Postmenopausal vs Premenopausal) | P07942, P14543, P54709, P52566, P04179, Q01844, Q5VT25, P43490, P19367, O00151, P01877, Q9H299, Q15424 |

# Optimal Hyper-parameter

The optimal hyper-parameters of each model were determined by Grid Search with 5 times and 10-fold Repeated k-Fold Cross- Validation (Supplementary Table 2).

**Supplementary Table 2: The optimal hyper-parameters of ML models determined by Grid Search**

| **Model** | **Optimal Hyper- Parameters** |
| --- | --- |
| AdaBoost | n_estimators =600, learning_rate= 0.01 |
| LightGBM | colsample_bytree = 0.7, max_depth = 9, n_estimators =600, num_leaves = 50, reg_alpha =0.005, subsample = 0.9 |
| XGBoost | reg_alpha=0.0005, nthread=1, scale_pos_weight=1, learning_rate = 0.03, gamma=0.4, subsample=0.9, colsample_bytree=0.8, min_child_weight=1, max_depth = 7, n_estimators = 700 |

# Evaluation Criteria

Supplementary Table 3: Classification matrix for calculating performance criteria

|  | | **Real** | | |
| --- | --- | --- | --- | --- |
|  |  | **Positive** | **Negative** | **Total** |
| **Predicted** | **Positive** | True positive (TP) | False positive (FP) | **TP+FP** |
|  | **Negative** | False negative (FN) | True negative (TN) | **FN+TN** |
|  | **Total** | **TP+FN** | **FP+TN** | **TP+TN+FP+FN** |

Accuracy = (TP+TN)/(TP+TN+FP+FN)

Sensitivity = TP/(TP+FN)

Specificity = TN/(FP+TN)

F1-score = (2*TP)/(2*TP+FP+FN)

AUC: The AUC is a performance metric that measures the area under the Receiver Operating Characteristic (ROC) curve, which is a graphical representation of a model's ability to distinguish between two classes (usually a positive class and a negative class) across different probability thresholds.

# Statistical analysis

Descriptive statistics for the proteins included in the model after the Lasso method in terms of tumor size (microscopic vs macroscopic), mymetrial invasion (<10% vs. >10%), grade (Grade I vs High Grade), and age (postmenopausal vs premenopausal) in EC patients within the scope of this study are given in Supplementary Tables 4.1-4.4.

**Supplementary Table 4.1:** Descriptive statistics for proteins included in the model after the Lasso method in terms of Tumor Size

| **Accession** | **Gene Name** | **Protein Name** | **Group** | | | | **p-value** |
| --- | --- | --- | --- | --- | --- | --- | --- |
|  |  |  | **Macroscopic (n=37)** | | **Microscopic (n=24)** | |  |
|  |  |  | **Mean±SD** | **Median (Min-Max)** | **Mean±SD** | **Median (Min-Max)** |  |
| P02730 | SLC4A1 | Band 3 anion transport protein | -0,56±1,98 | -0,66(-3,51-4,66) | 1,02±2,4 | 0,68(-2,68-5,6) | **0,032*** |
| P35555 | FBN1 | Fibrillin-1 | 1,36±1,53 | 1,17(-1,06-5,51) | 3,48±1,86 | 3,78(-0,27-7,16) | **<0,001*** |
| O43294 | TGFB1I1 | Transforming growth factor beta-1-induced transcript 1 protein | -2,55±1,5 | -2,78(-4,81-2,04) | -0,93±2,77 | -2,24(-4,31-4,97) | **0,042*** |
| P26447 | S100A4 | Protein S100-A4 | -0,13±1,29 | -0,19(-3,16-2,99) | 1,02±1,92 | 0,99(-3,89-4,27) | **0,007**** |
| P55001 | MFAP2 | Microfibrillar-associated protein 2 | -0,13±2,01 | -0,23(-4,34-4,47) | 1,82±1,53 | 2,34(-3,18-3,67) | **<0,001*** |
| P33991 | MCM4 | DNA replication licensing factor MCM4 | 0,37±1,05 | 0,1(-1,57-3,35) | 1,96±1,74 | 1,86(-1,27-5,44) | **<0,001*** |
| P24043 | LAMA2 | Laminin subunit alpha-2 | -2,85±1,42 | -2,49(-6,78--0,2) | -1,47±1,58 | -1,3(-5,81-1,61) | **<0,001*** |
| P02462 | COL4A1 | Collagen alpha-1(IV) chain | -0,79±1,16 | -0,78(-3,02-1,66) | 0,4±1,89 | 0,2(-2,64-4,25) | **0,009**** |
| Q16555 | DPYSL2 | Dihydropyrimidinase-related protein 2 | 2,05±1,26 | 2,23(-0,82-4,34) | 3,96±1,11 | 3,7(2,18-6,59) | **<0,001**** |
| Q02952 | AKAP12 | A-kinase anchor protein 12 | -1,16±1,47 | -1,21(-4,01-1,45) | 0,54±1,27 | 0,99(-2,25-2,03) | **<0,001*** |
| P39060 | COL18A1 | Collagen alpha-1(XVIII) chain | -0,11±1,26 | 0,11(-2,49-2,16) | 1,39±0,95 | 1,42(-0,38-3,84) | **<0,001**** |
| P22695 | UQCRC2 | Cytochrome b-c1 complex subunit 2, mitochondrial | 1,23±0,85 | 1,31(-0,94-3,02) | 2,08±1,03 | 2,17(-0,17-3,93) | **0,002**** |
| O43175 | PHGDH | D-3-phosphoglycerate dehydrogenase | 1,61±0,94 | 1,69(-0,2-3,78) | 0,73±1,03 | 0,47(-0,59-3,26) | **0,001*** |
| Q01844 | EWSR1 | RNA-binding protein EWS | -0,84±2,62 | -1,23(-5,27-4,95) | -2,96±1,69 | -3,64(-5,02-1,43) | **0,001*** |

*: Mann-Whitney U test; **: Independent Sample t-test

**Supplementary Table 4.2:** Descriptive statistics for proteins included in the model after the Lasso method in terms of Mymetrial Invasion

| **Accession** | **Gene Name** | **Protein Name** | **Group** | | | | **p-value** |
| --- | --- | --- | --- | --- | --- | --- | --- |
|  |  |  | **<10% (n=37)** | | **>10% (n=22)** | |  |
|  |  |  | **Mean±SD** | **Median (Min-Max)** | **Mean±SD** | **Median (Min-Max)** |  |
| Q9HC35 | EML4 | Echinoderm microtubule-associated protein-like 4 | 0,75±0,53 | 0,81(-0,68-1,72) | -0,41±0,85 | -0,48(-1,95-1,49) | **<0,001*** |
| P07305 | H1-0 | Histone H1.0 | 2,93±1,69 | 2,83(0,41-6,15) | 4,89±1,12 | 4,98(3,02-7,21) | **<0,001**** |
| Q13425 | SNTB2 | Beta-2-syntrophin | -1,15±1,23 | -1,16(-3,36-0,78) | 0,32±1,23 | 0,78(-1,87-2,07) | **<0,001**** |
| Q9UII2 | ATP5IF1 | ATPase inhibitor, mitochondrial | -0,43±1,23 | -0,45(-3,71-2,36) | 0,95±1,28 | 0,95(-1,49-3,36) | **<0,001**** |
| Q09028 | RBBP4 | Histone-binding protein RBBP4 | -0,23±1,4 | -0,08(-3,71-2,09) | -1,59±0,87 | -1,5(-3,11--0,04) | **<0,001**** |
| O75348 | ATP6V1G1 | V-type proton ATPase subunit G 1 | -2±1,27 | -1,72(-4,69-0,36) | -0,79±0,71 | -0,6(-2,2-0,13) | **<0,001**** |
| P14209 | CD99 | CD99 antigen | -0,59±0,96 | -0,77(-2,84-1,04) | 0,57±0,92 | 0,31(-0,83-2,17) | **<0,001*** |
| P61289 | PSME3 | Proteasome activator complex subunit 3 | 0,07±1,12 | 0,17(-2,28-2,66) | -1,16±0,88 | -1,1(-3,13-0,25) | **<0,001**** |
| Q9NQW7 | XPNPEP1 | Xaa-Pro aminopeptidase 1 | -0,39±0,78 | -0,4(-2,44-1,12) | -1,41±1,25 | -1,49(-3,77-1,32) | **<0,001**** |
| Q93099 | HGD | Homogentisate 1,2-dioxygenase | -1,54±1,26 | -1,57(-3,91-0,81) | -2,88±1,08 | -2,74(-5,22--0,37) | **<0,001**** |
| P08572 | COL4A2 | Collagen alpha-2(IV) chain | 0,49±1,04 | 0,61(-1,58-3,47) | 1,87±1,33 | 1,85(-0,17-4,28) | **<0,001**** |
| P22695 | UQCRC2 | Cytochrome b-c1 complex subunit 2, mitochondrial | 1,25±0,89 | 1,16(-0,94-3,16) | 2,14±0,98 | 2,15(-0,17-3,93) | **<0,001**** |
| P23921 | RRM1 | Ribonucleoside-diphosphate reductase large subunit | -5,53±1,3 | -5,74(-7,81--1,75) | -4,08±1,21 | -4,37(-6,14--1,39) | **<0,001**** |
| Q9NRN5 | OLFML3 | Olfactomedin-like protein 3 | -1,32±1,33 | -1,37(-3,35-2,18) | 0,2±2,18 | -0,02(-2,77-3,65) | **0,012*** |
| P54108 | CRISP3 | Cysteine-rich secretory protein 3 | -3,96±1,5 | -4,05(-6,15-0,23) | -2,48±1,78 | -3,01(-4,76-1,85) | **<0,001**** |
| Q5VTU8 | ATP5F1EP2 | ATP synthase subunit epsilon-like protein, mitochondrial | 1,37±1,09 | 1,56(-1,44-3,2) | 2,42±1,05 | 2,49(-0,97-3,74) | **0,001*** |
| P62328 | TMSB4X | Thymosin beta-4 | 1±2,59 | 1(-3,2-5,3) | 3,08±1,45 | 2,86(0,03-5,6) | **<0,001**** |
| Q01844 | EWSR1 | RNA-binding protein EWS | -1,02±2,47 | -1,23(-4,56-4,95) | -2,92±1,77 | -3,25(-5,27-1,41) | **0,002*** |

*: Mann-Whitney U test; **: Independent Sample t-test

**Supplementary Table 4.3:** Descriptive statistics for proteins included in the model after the Lasso method in terms of Grade I-High Grade

| **Accession** | **Gene Name** | **Protein Name** | **Group** | | | | **p-value** |
| --- | --- | --- | --- | --- | --- | --- | --- |
|  |  |  | **Grade I (n=37)** | | **High Grade (n=4)** | |  |
|  |  |  | **Mean±SD** | **Median (Min-Max)** | **Mean±SD** | **Median (Min-Max)** |  |
| Q9BXP5 | SRRT | Serrate RNA effector molecule homolog | 0,9±0,71 | 0,9(-0,54-2,45) | 2,23±0,34 | 2,24(1,82-2,63) | **0,001*** |
| P49591 | SARS1 | Serine--tRNA ligase, cytoplasmic | -0,13±0,94 | -0,21(-1,89-1,69) | -2,01±0,75 | -1,86(-3,06--1,28) | **0,001*** |
| O75367 | MACROH2A1 | Core histone macro-H2A.1 | 2,83±0,75 | 2,77(1,1-4,14) | 4,11±0,26 | 4,05(3,88-4,44) | **<0,001*** |
| Q5SSJ5 | HP1BP3 | Heterochromatin protein 1-binding protein 3 | 2,37±0,82 | 2,31(-0,01-4) | 3,62±0,25 | 3,68(3,27-3,86) | **0,004*** |
| P49913 | CAMP | Cathelicidin antimicrobial peptide | 1,19±1,74 | 0,78(-1,37-4,89) | -1,69±0,37 | -1,7(-2,07--1,29) | **<0,001*** |
| P30086 | PEBP1 | Phosphatidylethanolamine-binding protein 1 | 3,17±1,14 | 3,49(0,81-5) | 4,84±0,08 | 4,83(4,75-4,95) | **0,001*** |
| P00491 | PNP | Purine nucleoside phosphorylase | 0,96±0,88 | 0,85(-1,43-2,41) | -0,54±0,71 | -0,33(-1,54-0,03) | **0,001*** |
| O75489 | NDUFS3 | NADH dehydrogenase [ubiquinone] iron-sulfur protein 3, mitochondrial | 0,09±0,79 | 0,02(-1,57-2,48) | -0,98±0,66 | -1,15(-1,58--0,04) | **0,014*** |
| Q03060-9 | CREM | cAMP-responsive element modulator | -1,48±1,65 | -1,04(-5,22-0,62) | 0,31±0,15 | 0,34(0,12-0,45) | **0,007*** |

*: Mann-Whitney U test

**Supplementary Table 4.4:** Descriptive statistics for proteins included in the model after the Lasso method in terms of Postmenopausal-Premenopausal

| **Accession** | **Gene Name** | **Protein Name** | **Group** | | | | **p-value** |
| --- | --- | --- | --- | --- | --- | --- | --- |
|  |  |  | **Postmenopausal (n=41)** | | **Premenopausal (n=18)** | |  |
|  |  |  | **Mean±SD** | **Median (Min-Max)** | **Mean±SD** | **Median (Min-Max)** |  |
| P07942 | LAMB1 | Laminin subunit beta-1 | 1,58±0,61 | 1,5(0,59-3,13) | 2,59±0,76 | 2,63(0,8-3,82) | **<0,001**** |
| P14543 | NID1 | Nidogen-1 | 0,8±0,83 | 0,57(-0,59-2,4) | 1,81±0,71 | 2(0,27-2,93) | **<0,001**** |
| P54709 | ATP1B3 | Sodium/potassium-transporting ATPase subunit beta-3 | -0,82±1,27 | -0,37(-4,84-0,95) | 0,16±0,79 | 0,43(-1,59-1,15) | **0,002*** |
| P52566 | ARHGDIB | Rho GDP-dissociation inhibitor 2 | 0,25±1,59 | 0,47(-2,45-5,16) | 1,76±1,16 | 1,66(-0,2-4,35) | **0,001**** |
| P04179 | SOD2 | Superoxide dismutase, mitochondrial | 1,02±1,17 | 0,97(-1,16-4,33) | 2,11±0,87 | 2,12(0,67-3,48) | **<0,001**** |
| Q01844 | EWSR1 | RNA-binding protein EWS | -1,24±2,41 | -1,49(-5,27-4,95) | -2,84±2,04 | -3,92(-5,02-1,41) | **0,009*** |
| Q5VT25 | CDC42BPA | Serine/threonine-protein kinase MRCK alpha | -2,37±1,32 | -2,25(-6,77-0,07) | -1,21±1,15 | -1,53(-2,38-2,3) | **0,003*** |
| P43490 | NAMPT | Nicotinamide phosphoribosyltransferase | 1,73±1 | 1,86(-0,6-3,35) | 0,92±0,89 | 1,13(-0,73-2,38) | **0,005**** |
| P19367 | HK1 | Hexokinase-1 | 2,06±0,64 | 2,19(0,11-3,29) | 2,8±0,73 | 2,94(1,49-4,67) | **0,001*** |
| O00151 | PDLIM1 | PDZ and LIM domain protein 1 | 1,83±1,09 | 1,96(-0,63-4,06) | 2,95±1,31 | 3,07(0,12-5,09) | **0,001**** |
| P01877 | IGHA2 | Immunoglobulin heavy constant alpha 2 | -1,25±2,05 | -1,02(-5,17-3,89) | -2,93±2 | -3,43(-5,54-1,17) | **0,005**** |
| Q9H299 | SH3BGRL3 | SH3 domain-binding glutamic acid-rich-like protein 3 | 2,11±0,89 | 1,87(-0,02-4,15) | 3,13±1,23 | 3,12(1,11-5,33) | **0,003*** |
| Q15424 | SAFB | Scaffold attachment factor B1 | 0,7±0,85 | 0,75(-2,25-2,32) | -0,3±1,42 | 0,22(-2,7-1,36) | **0,012*** |

*: Mann-Whitney U test; **: Independent Sample t-test
